# Risk of healthcare visits from influenza in subjects with diabetes and impacts of early vaccination

**DOI:** 10.1136/bmjdrc-2023-003841

**Published:** 2024-08-06

**Authors:** Ronald Horswell, San Chu, Addison E Stone, Daniel Fort, Gabriel Uwaifo, Vivian A Fonseca, Elizabeth B Norton

**Affiliations:** 1 Pennington Biomedical Research Center, Baton Rouge, Louisiana, USA; 2 Department of Microbiology & Immunology, Tulane University School of Medicine, New Orleans, Louisiana, USA; 3 Ochsner Center for Outcomes and Health Services Research, New Orleans, Louisiana, USA; 4 Department of Endocrinology, Diabetes, and Metabolism, Ochsner Medical Center, New Orleans, Louisiana, USA; 5 Medicine, Tulane University, New Orleans, Louisiana, USA

**Keywords:** Diabetes Mellitus, Type 2, Vaccination, Infectious Disease Medicine

## Abstract

**Introduction:**

The objective of this study was to determine the burden of influenza disease in patients with or without diabetes in a population of American adults to understand the benefits of seasonal vaccination.

**Research design and methods:**

We performed a retrospective cohort study using electronic medical records totaling 1,117,263 from two Louisiana healthcare providers spanning January 2012 through December 2017. Adults 18 years or older with two or more records within the study period were included. The primary outcome quantified was influenza-related diagnosis during inpatient (IP) or emergency room (ER) visits and risk reduction with the timing of immunization.

**Results:**

Influenza-related IP or ER visits totaled 0.0122–0.0169 events per person within the 2013–2016 influenza seasons. Subjects with diabetes had a 5.6-fold more frequent influenza diagnosis for IP or ER visits than in subjects without diabetes or 3.7-fold more frequent when adjusted for demographics. Early immunization reduced the risk of influenza healthcare utilization by 66% for subjects with diabetes or 67% for subjects without diabetes when compared with later vaccination for the 2013–2016 influenza seasons. Older age and female sex were associated with a higher incidence of influenza, but not a significant change in risk reduction from vaccination.

**Conclusions:**

The risk for influenza-related healthcare utilization was 3.7-fold higher if patients had diabetes during 2013–2016 influenza seasons. Early immunization provides a significant benefit to adults irrespective of a diabetes diagnosis. All adults, but particularly patients with diabetes, should be encouraged to get the influenza vaccine at the start of the influenza season.

**WHAT IS ALREADY KNOWN ON THIS TOPIC**

- Diabetes is a risk factor for adverse health outcomes; however very few studies have ever tried to quantify the risk for severe influenza illness in patients with diabetes as well as calculating how early influenza vaccination reduces this risk.

**WHAT THIS STUDY ADDS**

- In this retrospective cohort, patients with diabetes in Louisiana, USA had 3.7-fold more frequent influenza diagnosis visits. Immunization early in the influenza season reduced the risk of influenza-related healthcare utilization by 66–78% in subjects with or without diabetes.

**HOW THIS STUDY MIGHT AFFECT RESEARCH, PRACTICE OR POLICY**

- This study supports that all adults, especially those with diabetes, should be encouraged to get the influenza vaccine early in the influenza season.

## Introduction

 Diabetes is recognized as a risk factor for adverse health outcomes due to infection; however, the increased burden of disease associated with patients with diabetes is unclear. Few studies have attempted to compare the burden of seasonal influenza in adults with and without diabetes,^1^,^4^ and no studies in the USA have evaluated both sexes and/or racially diverse study populations.

Influenza vaccination is recommended by the Centers for Disease Control and Prevention (CDC) for everyone 6 months and older based on studies evaluating vaccine efficacy, with adults older than 65 years or individuals with certain chronic health conditions (eg, diabetes) noted for higher risk of developing serious influenza complications.^5^,^7^ This recommendation has been made based on the morbidity and mortality rates in these populations, as well as the burden of healthcare-associated costs quantified.^8^ Quantifying adverse health outcomes in the context of vaccine utilization, particularly in populations with comorbidities such as diabetes, remains an important public health and clinical need. In light of the recent SARS-CoV-2 pandemic, several studies have investigated the severity of influenza seasons and the associations between SARS-CoV-2 and influenza vaccination on hospitalization and morbidity related to COVID-19, though not in the Northern Hemisphere or in patients with diabetes.^9^,^11^ Recently, high-dose inactivated influenza vaccination has been recommended in adults over the age of 65 based on older adults mounting a less robust immune response to vaccination,^12 13^ though the effectiveness of this updated vaccine on patients with diabetes has yet to be investigated.

The prevalence of diabetes is increasing across the USA, particularly in the southern states.^14^ Quantification of influenza-attributed healthcare utilization and the benefits of vaccination can help drive evidence-based healthcare. The goal of this study was to determine the diabetes-attributable risk of influenza using electronic records from two provider organizations in Louisiana spanning the 2012–2016 influenza seasons. We also used this data to understand the relative risk reduction from seasonal vaccination using two methodologies.

## Research design and methods

### IRB approval, data extraction and inclusion criteria

This retrospective cohort study was performed using data extracted from the REACHnet Common Data Model (CDM) from Tulane Health and Ochsner Medical Center provider organizations.^15^,^17^ Data was extracted only for subjects over the age of 18 with two or more ambulatory clinic visits in the time span of January 1, 2012, through December 31, 2017. A diabetes determination for each subject was determined by two diabetes diagnoses at least 30 days apart. Eligibility for inclusion in the analyses was assessed separately for each influenza season; that is, an individual eligible for inclusion in, say, the 2014–2015 influenza season analysis might not be eligible for inclusion in the 2015–2016 analysis. For each influenza season eligibility was based on the occurrence of outpatient clinic visits (not including emergency department (ED) visits), with eligibility requiring that an individual have both (1) an outpatient clinic visit prior to September 1 (eg, prior to September 1, 2014 for the 2014–2015 influenza season) and (2) an outpatient clinic visit after the following March 31, although death during the influenza season negated the second requirement. The above eligibility requirement was to help ensure that included individuals’ major health events would be represented in the available data. In addition to the above, a third eligibility requirement was that an individual have a history of encounters (represented in the available data) that extended back at least 1-year prior to the beginning of each influenza season. The reason for this third requirement was to enable the calculation of (1) the Comorbidity Index and (2) the recent history of ED visits and inpatient stays, both of which are terms used in the statistical analysis for risk-adjustment purposes. The Comorbidity Index was constructed explicitly for purposes of risk adjustment for this project’s outcomes. That index allowed for the possibility that any prior diagnosis potentially could be related to the subsequent probability of an adverse influenza season event. Our objective in creating the index was to avoid relying on prior assumptions regarding which prior diagnoses might be related to the target outcomes of interest. However, in any event, the recent history of ED visits and inpatient stays was a much more potent risk adjustment factor than the Comorbidity Index.

Four different definitions of outcomes were used in analyses using International Classification of Disease (ICD)-9 and ICD-10 diagnostic codes (see online supplemental table S1) including iped_infpneu (inpatient and ED stay events carrying a diagnosis of either influenza or pneumonia), iped_resp (inpatient and ED stay events carrying a diagnosis of influenza, pneumonia, or some respiratory illness), iped_all (all-cause inpatient and ED stay events) and ip_all (all inpatient stay events), differing in the degree to which they are directly related to influenza. To be considered two distinct clinical episodes, events had to occur a minimum of 7 days apart.

### Publicly availability data sets

Influenza-like illness (ILI) census data collected through the USA. Outpatient Influenza-like Illness Surveillance Network (ILINet) by week from Louisiana or USA was accessed using https://wwwn.cdc.gov/ilinet/ for ILI events by influenza season.

### Determination of influenza diagnosis risk

Events per person for iped_infpneu by influenza season were calculated for subjects by comorbidity status (eg, diabetes or without diabetes) or for subjects without diabetes adjusted for comorbidities and demographics to those with diabetes. We used 95% CIs and a 0.05 statistical significance threshold. The SEs were generated by bootstrap methods; that is, sampling with replacements of subjects. The CIs are somewhat asymmetric, as the CIs were generated for the estimates of log(relative risk) and then exponentiated.

### Estimation of risk reduction with immunization

Immunization status was derived from either an immunization event, as a medication prescription or administration, or as a procedure. As the Research Action for Health Network (REACHnet) CDM does not include data from retail pharmacies, a common site of influenza vaccination, or claims data, the analytic cohort was limited to only patients with observed vaccination by one of the REACHnet-contributing health systems. A foundational assumption of this analysis is that each patient is vaccinated for influenza only once a year and therefore may only be considered reliably unvaccinated prior to their observed influenza vaccination. Relative risk estimations were then performed using the following methodologies.

#### Methodology A (or model 1)

A complete description of the methodology used for this model is available in (online supplemental methods). Briefly, Method A functions by defining two groups of vaccinated people for each influenza season of interest based on the timing of vaccination within the influenza season, either before or after a certain “split date” of January 1. Based on these two groups, matched pairs are chosen from one member of each group using gender, race (black, white, other), age (average age difference in the pair was 3.7 years), # of ED visits and hospitalization in the year prior (eg, 0, 1, 2, 3, 4+ visit), and an influenza-specific Comorbidity Index based on data prior to influenza season. The latter was created by estimating the marginal risk contributions associated with each three-digit ICD-9 and ICD-10 code. The resulting scaled risk values ranged from 0 to 8, with an SD=0.36. A matching algorithm again was used to minimize differences in index values within matched pairs. The average within-pair risk difference was 0.17. It was required that neither member of a matched pair had any outcome event between September 1 and 15 days after the immunization date of the Group 1 member of the pair. Estimation of vaccination effects was analyzed by appropriately comparing outcomes between Group 1 and Group 2 matched subjects over a time span when the Group 1 subject in each matched pair had been vaccinated, but the Group 2 subject had not yet been vaccinated. The event rate comparison was made using a Poisson regression model, with SEs estimated by bootstrap methods.

#### Methodology B (or model 2)

Methodology A has certain potential conceptual limitations from: (1) possible differences in vaccination effectiveness across months within an influenza season, and (2) the possibility that the timing of vaccination is related, in unknown ways, to outcomes risk. Methodology B was designed to mitigate the effects of such bias. Methodology B did not use overt matching, instead it involved first stratifying subjects based on gender, race (black, white, other) and age group (18–35, 36–45, 46–60, 61–76, 76+ years) variables. The statistical modeling then essentially compares the outcomes of those vaccinated to those unvaccinated in each month of the influenza season and generates an aggregated (across months) overall vaccination effect. Each subject in a particular influenza season contributes to both unvaccinated person-years for each month pre-vaccination and also to the vaccinated person-years for each month post-vaccination. A negative binomial model was then used to estimate the above vaccination effects. In the modeling, the data were weighted by strata such that in each month, the vaccinated time (in person-years) had the same (weighted) demographic distribution as the unvaccinated time. This included (1) a binary variable identifying the month (to adjust for monthly differences in outcomes levels), (2) a binary variable indicating whether or not the individual was newly vaccinated (as opposed to having been vaccinated in previous months) (included to control for possibility that the timing of vaccination was motivated by unknown factors), (3) the number of ED visits and hospitalization in the year prior to the beginning of the influenza season (0, 1, 2, 3, 4+ visits), and (4) the influenza-specific Comorbidity Index described earlier. SEs were estimated via bootstrap methods.

### Statistics and graphs

Data management and statistical analyses were carried out using Stata (V.14, StataCorp, College Station, Texas, USA). The type I error threshold was set at 5%. For both models, the SEs were obtained by bootstrap methods, and CIs and p values are based on those bootstrap-derived SEs. Graphs were made using Stata V.14, GraphPad Prism V.9.5.1 and Adobe Illustrator V.27.2. Model images and graphical abstract were made using BioRender.com software.

### Funding

NIH/NIGMS IDeA-CTR U54 GM104940, PCORI Award RI-LPHI-01-PS1, NIH/NIAID/R01AI166682, and NIH/NCI U54CA260581 provided funding for this study. The content of this publication is solely the responsibility of the authors and does not necessarily represent the official view of the NIH and the NIH had no role in the data analysis or manuscript preparation.

### Data and resource availability

The data that support the findings of this study are available from REACHnet but restrictions apply to the availability of these data, which were used under license for the current study and therefore are not publicly available. Data are however available from the authors on reasonable request and with permission of REACHnet.

## Results

### Subjects identified from Louisiana healthcare providers could be stratified by influenza season (2012–2016) and history of diabetes

**Figure 1:**
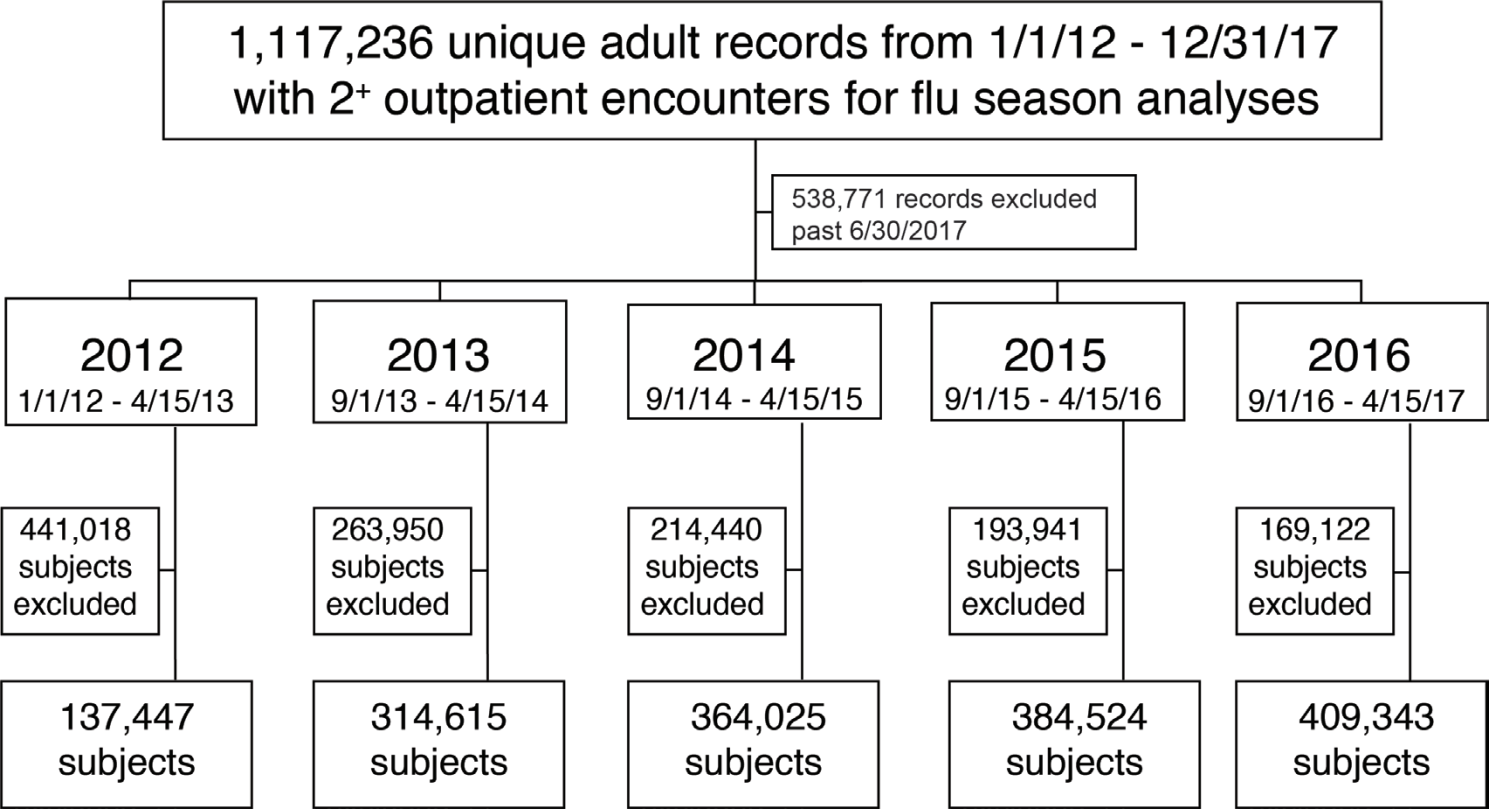
Overview of subject records used in this study by influenza year. 1,117,236 unique individuals from patients aged 18 years and older were extracted from the Research Action for Health Network Common Data Model for two provider organizations in Louisiana (Tulane Health, Ochsner Medical Center). Extraction criteria included aged 18+ years and two or more ambulatory clinic visits during January 1, 2012, to December 31, 2017. These records were further defined within each yearly influenza season, and excluded if they did not have two or more outpatient clinic visits spanning at least 90 days prior to the start of the influenza season (eg, September 1 to April 15) in order to identify a population of subjects attached to the provider systems included in this study. Subjects were included in multiple influenza seasons if meeting inclusion criteria.

We pulled 1,117,236 unique adult records from two provider organizations, Tulane Health and Ochsner Medical Center, in Louisiana spanning January 2012 to the end of 2017. This time period covered five influenza seasons including any disease activity between September 1 and April 15 (2012–2016, figure 1). Subject records were included if two or more outpatient clinic encounters were recorded during or within 90 days of each influenza season. Between 137,447 and 409,343 subjects were captured using this method per season (figure 1). Patients with diabetes were identified as 30–35% of all subjects within the four influenza seasons (online supplemental table S2). The subject race was primarily identified as white (62–70%) and black (28–36%) with median ages between 48 and 64 years old using data from each patient group and season. Patients with diabetes tended to be older (46–49%>65 years with diabetes vs 20–24%>65 years without diabetes) and have an increased proportion of male sex (56% women with diabetes vs 64–67% women without diabetes).

### Rates of influenza-related outcomes by diagnosis of diabetes

**Figure 2:**
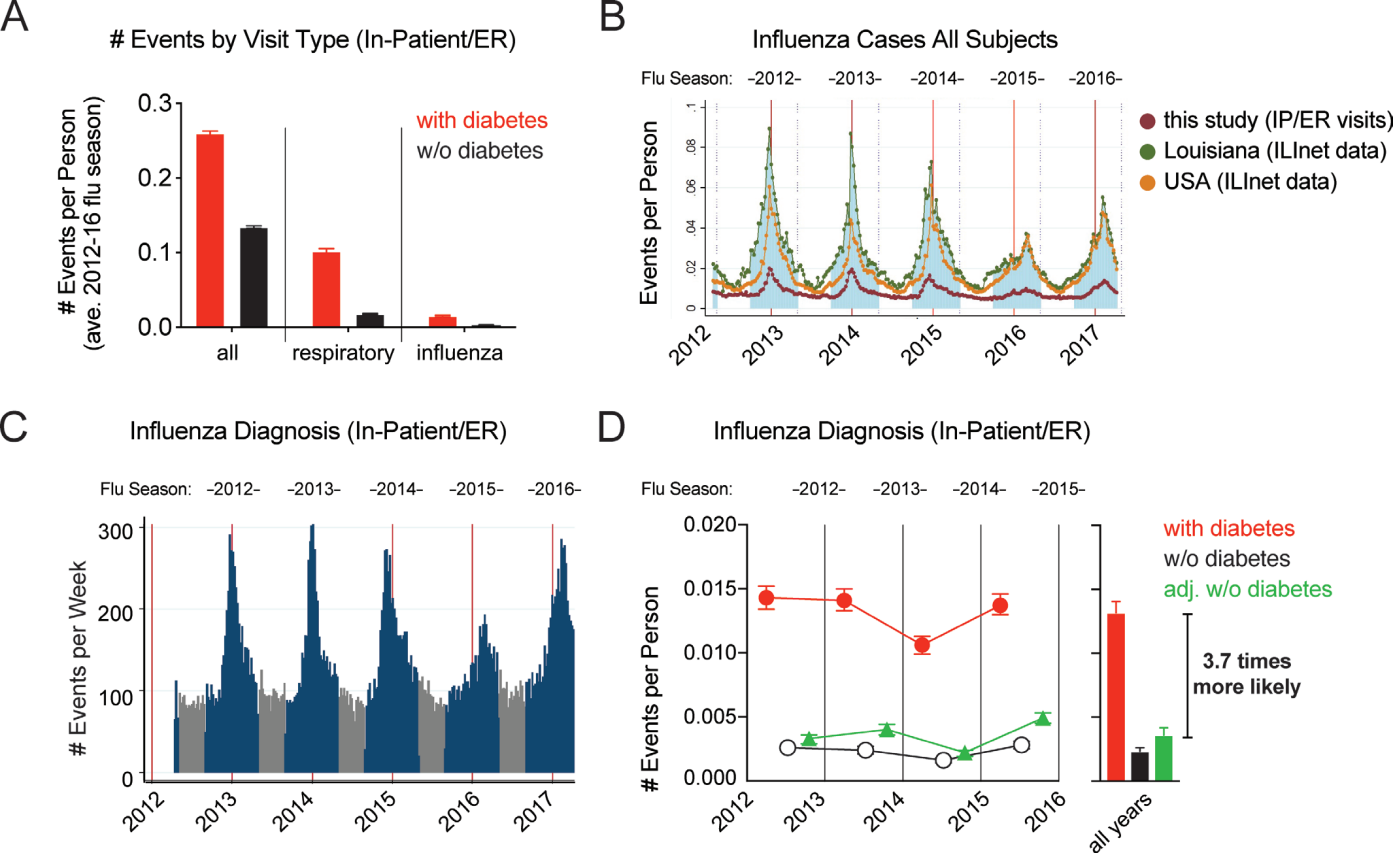
Seasonality of influenza-related pneumoniae events and higher level of healthcare utilization in subjects with diabetes between 2012 and 2016 influenza seasons. (**A**) Events per person averaged per 2012–2016 influenza season for all inpatient/ER visits (iped_all), respiratory (iped_resp), or influenza (iped_infpneu) irrespective of patient population (unadjusted). (**B**) Events per person (# events/subjects reported by week) by week for influenza-related healthcare utilization in this study (influenza, red line); or publicly available influenza-like illness ILINet census data by week from Louisiana (green line) or USA (orange line). Axis begins on January 1. Respective influenza season is shown with blue shading and top label and all data is unadjusted. (**C**) Histogram of influenza diagnosis events per week over time (unadjusted data) with influenza season (blue, September to April) or non-influenza season (gray, May to August) shown. (**D**) Influenza diagnosis events per subject by influenza season in subjects with (red symbols) or without (blue symbols) a previous diabetes diagnosis. Green symbols show results for subject without diabetes adjusted to be standardized to the demographic (age, sex, race) and comorbidity characteristics of the subjects with diabetes. Error bars on line symbols indicate 95% CIs. Bars indicate all years mean with error bars at SEM. ER, emergency room; ILINet, Influenza-like Illness Surveillance Network; IP, inpatient.

Clinical codes for inpatient or emergency room (ER) visits were analyzed for all, respiratory or influenza events as evidence of healthcare utilization over the study period (figure 2A, online supplemental figure S1). Disease classification codes for both influenza diagnosis and pneumonia were used to define influenza-related events (online supplemental table S1) to capture both viral pneumoniae, high-risk of secondary bacterial pneumoniae,^18^ and overcome any errors in pneumonia coding entries. This included the 2015 influenza season during the ICD-9 to ICD-10 coding change, which likely contributed to lower influenza events when a stricter coding definition was used (online supplemental figure S1). The number of influenza events in this study followed seasonal patterns observed with census data for state and country ILI (ILINET data for USA and Louisiana, figure 2B,C) or other coding definitions (online supplemental figure S1). Events from the 2012 influenza season were not evaluated in order to both confirm the diagnosis of diabetes and to create an adjusted control group for demographic and comorbidities. Across seasons, influenza events were higher in subjects with diabetes (0.0106–0.0143 events per person) than subjects without diabetes (0.0022–0.0049) and adjusted subjects without diabetes (0.0016–0.0028, figure 2D, online supplemental table S2). Thus, subjects with diabetes had a 5.6-fold higher rate of influenza-related events than subjects without diabetes or a 3.7-fold higher rate with adjustment for other factors (age, sex, race, etc) during the influenza season.

### Benefits of influenza vaccination in patients with or without diabetes

**Figure 3:**
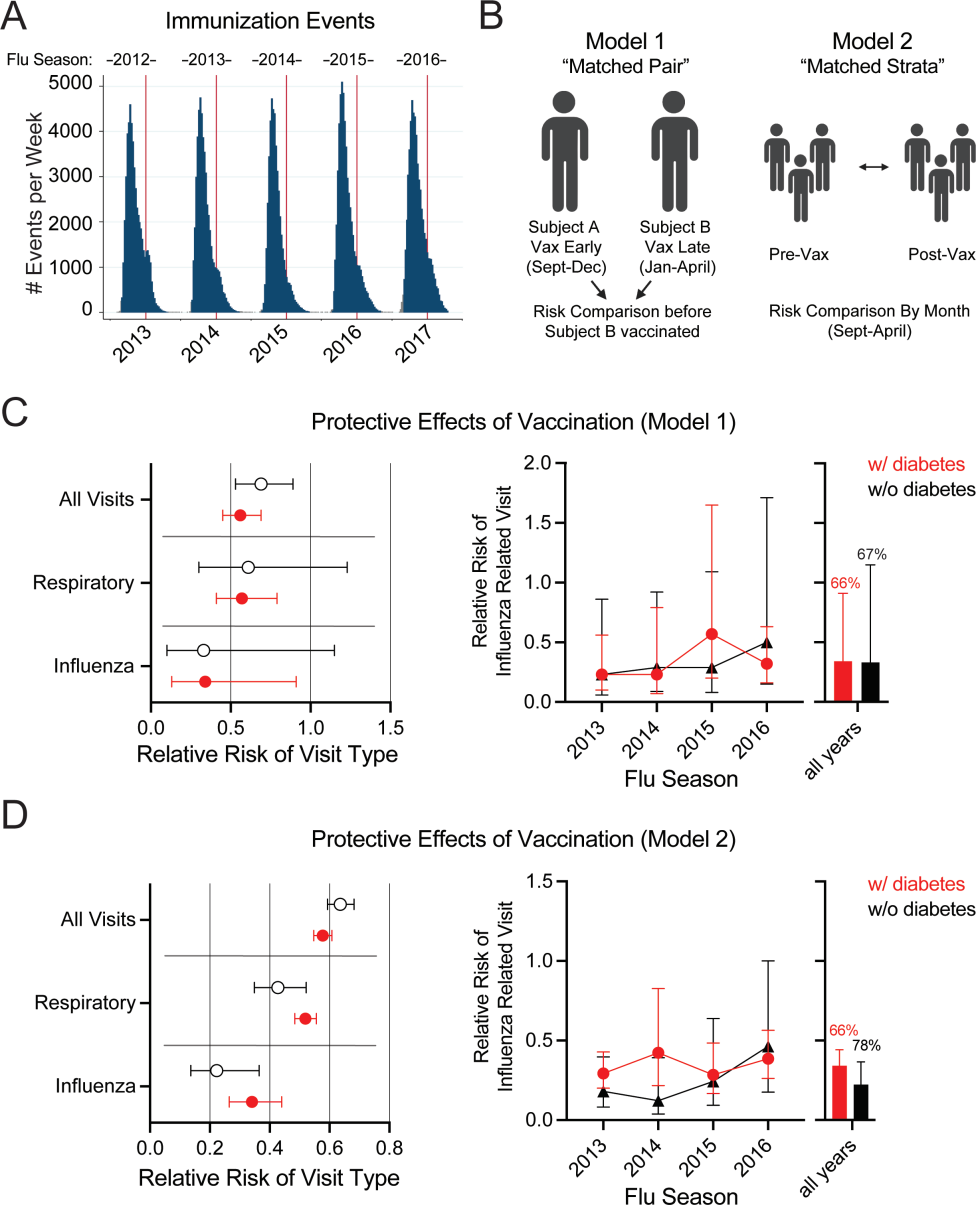
Vaccination results in a decreased risk of healthcare utilization regardless of diabetes diagnosis. (**A**) Immunization events in study cohort during 2012–2016 influenza seasons (top label). (**B**) Schematic representation of Model 1 and Model 2 used for study analyses. Subjects in Model 1 were matched based on demographics (gender, race, age), # of emergency department and inpatient stays the previous year, comorbidities and vaccination early (September to December) or late (January to April) in the influenza season. Subjects in Model 2 were placed in matched by month (gender, race, age group) by month and vaccination status. (**C**) Relative risk analyses of all (iped_all), respiratory (iped_resp), and influenza-related healthcare utilization (iped_infpneu) in immunized versus non-immunized subjects using Model 1. (**D**) Relative risk analyses of all, respiratory, and influenza-related healthcare utilization in immunized versus non-immunized subjects using Model 2. Error bars on symbols indicate 95% CIs. Bars indicate all years mean with error bars at SEM.

Immunization history was determined for available records for each complete influenza season (2012–2016; online supplemental figure S2, figure 3A). Immunization outside of the provider system frequently occurred during the study period through pharmacy-led public health interventions (eg, Walgreens,^19^) preventing a direct comparison of immunized and non-immunized subjects. To overcome this issue, we used two new methods for examining vaccine protection by influenza season using only subjects with immunization events. In the first model, ‘matched pair’, we compared the risk of influenza events between subjects matched for demographics and comorbidities with one subject vaccinated early in the season (before January 1) and the other vaccinated later (anytime January 1 to April 15; figure 3B; online supplemental figure S3). This model does not assume consistent risk for influenza infection by month of an influenza season but does assume that subjects are randomly immunized during the season. In the second model, “matched by month”, vaccinated subjects were compared with not yet vaccinated subjects (eg, subjects who had a recorded immunization later in the influenza season) within a month’s time to help account for elevated risk within specific populations (figure 3B; online supplemental figure S3). In both models, we found a reduction in the relative risk of healthcare utilization with immunization for all causes (not respiratory or influenza-specific), respiratory, or influenza-specific inpatient or ER visits (figure 3C,D). The reduction of risk was greatest for influenza diagnosis events with 43–88% estimated reduction depending on the year. Overall influenza seasons using Model 1, vaccination resulted in a 66% reduction in those with diabetes and a 67% reduction in those without diabetes (figure 3C, online supplemental table S3). Using Model 2, there was a 66% reduction in those with diabetes and a 78% reduction in those without diabetes (figure 3D, online supplemental table S3). There was no difference by diagnosis of diabetes indicating vaccination provided similar levels of protection to patients with diabetes and without, even though the overall number of influenza-attributed diagnoses was higher with diabetes. Subjects with diabetes had higher influenza events compared with unvaccinated subjects without diabetes irrespective of immunization status (online supplemental figure S3B).

### Influence of age and sex on influenza diagnosis and risk reduction with vaccination

**Figure 4:**
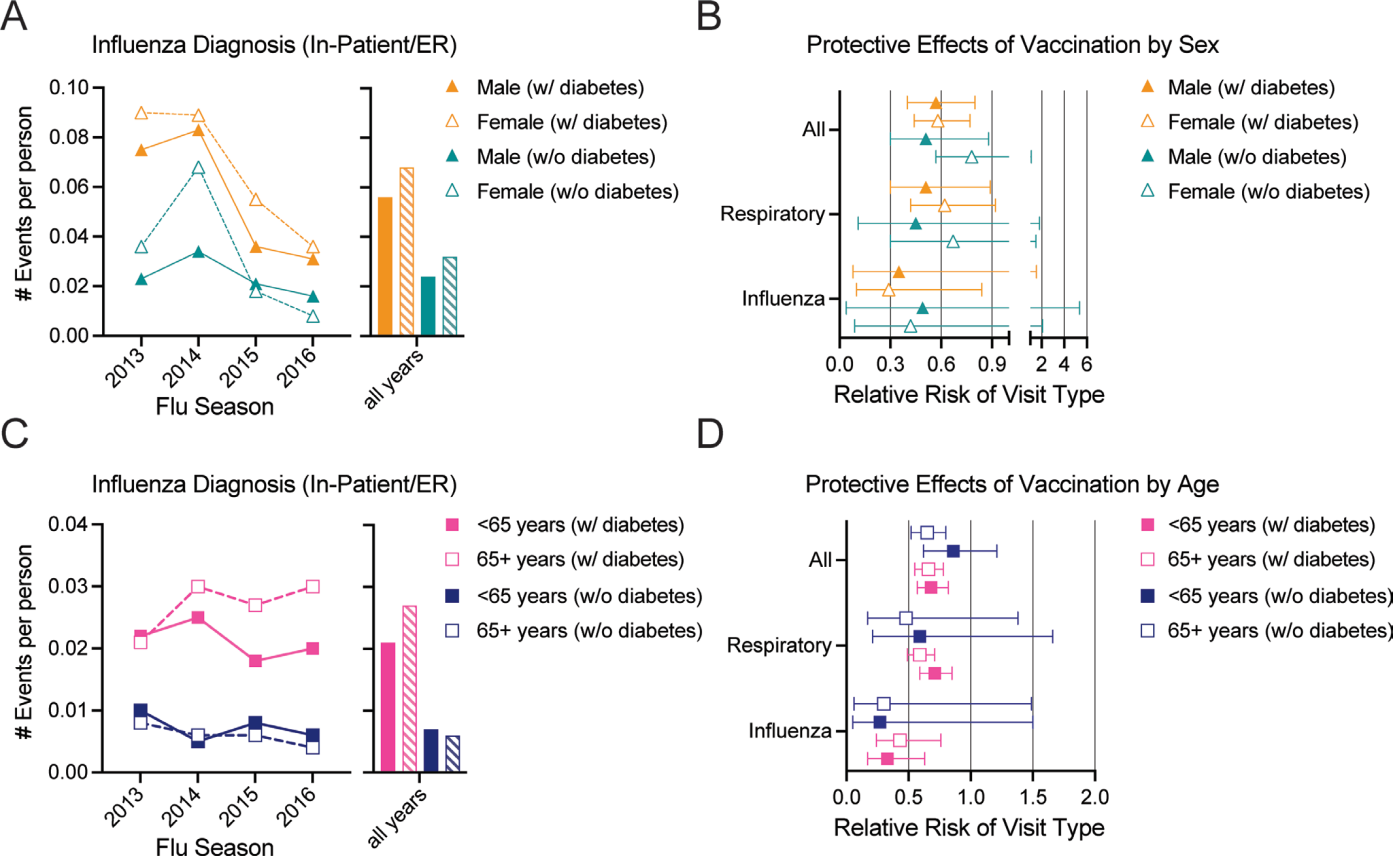
Age and sex differences in total healthcare events and relative risk using Model 2. (**A**) Influenza diagnosis events per person in male (closed symbol) and female (open symbol/hashed bar) subjects with (orange) or without (teal) diabetes across 2012–2016 influenza seasons and averaged for all years. (**B**) Relative risk analyses of all (iped_all), respiratory (iped_resp), and influenza-related healthcare utilization (iped_infpneu) in male and female subjects. (**C**) Influenza diagnosis events per person in adults <65 years (closed symbol) or 65+ years (open symbol/hashed bar) subjects with (pink) or without (dark blue) diabetes across 2012–2016 influenza seasons and averaged for all years. (**D**) Relative risk analyses of all (iped_all), respiratory (iped_resp), and influenza-related healthcare utilization (iped_infpneu) in adults <65 years or 65+ years. No significance was observed by patient and age or sex groups. Error bars on symbols indicate 95% CIs. Bars indicate all years mean with error bars at SEM. ER, emergency room.

We observed higher levels of influenza-related healthcare utilization in subjects with diabetes, irrespective of age or sex (figure 4). However, women and subjects 65+ years typically experienced the most influenza events, though seasonal differences were evident (figure 4A and C). The risk reduction from vaccination was present, but not significantly different within patient populations by sex or age for influenza seasons analyzed (2013–2016) (figure 4B and D, online supplemental figures S4 and S5).

## Discussion

This study defines influenza-attributed healthcare utilization in the first retrospective cohort study using electronic medical records in the USA. The study population used included diverse demographic profiles from Louisiana adults during 2012–2016 influenza seasons in two provider systems. We found that those with diabetes in Louisiana were 3.7 times more likely to have an influenza diagnosis at inpatient or ER visits than those without diabetes adjusted for similar demographic and comorbidities. Our data agrees with a recent meta-analysis of observational studies for higher levels of influenza complications in patients with diabetes.^20^

Influenza vaccination has been previously shown to prevent influenza infection and improve health outcomes and is recommended for all adults.^21^ We used two models to investigate the relative risk of healthcare utilization when individuals had an immunization record using either matched pairs or matched by month comparing risk before and after vaccination by early (pre-January) or late (January+) vaccination or by month during the influenza season. We found that regardless of diagnosis with diabetes, early vaccination protected from respiratory (41–75%), and influenza (43–82%) diagnosis for inpatient or ER visits across all influenza seasons. This matches influenza vaccine efficacy reported by the CDC, which ranged from 19% to 52% efficacy during this time period using multiple networks including laboratory-confirmed analyses in specific patient or hospital samples or randomized clinical trials.^22^ In addition, these comparisons provide an advanced strategy to avoid healthy user bias or handle measurements of vaccination outcomes when provider records for an unvaccinated population are unclear due to pharmacy-led public health vaccine campaigns. Risk reduction in those with and without diabetes was comparable; however, based on the~3.7-fold increase in healthcare utilization events during the influenza season, people with diabetes should still be targeted for early vaccination as there will be a~3.7-fold larger reduction of these influenza-associated events as well.

Several studies have examined the relationships between health status (eg, presence of chronic conditions) on susceptibility to serious influenza-related adverse outcomes using medical records. For example, using data from Canada, Lau *et al* concluded that diabetes increased the risk of serious influenza-related outcomes^3^ and Schanzer *et al* found associations between certain comorbidities and the risk of influenza-related adverse outcomes.^4^ In an analysis covering nearly two decades, Neuzil *et al* found an increased risk of adverse cardiovascular outcomes during influenza seasons among Medicaid enrollees in Tennessee.^1^

Other studies have combined data from various sources to compare the severity of outcomes across influenza seasons. Biggerstaff *et al*, for example, used data from several sources to estimate what they called intensity thresholds, which were then used to create a scale of influenza season severity.^9^ Studies also have examined the relationship between influenza vaccination and health outcomes during influenza seasons. Russo *et al* examined the relationship between influenza vaccination status and outcomes in Italy and also estimated the degree to which influenza vaccination may increase the level of protection from COVID-19 beyond the protection afforded by COVID vaccination itself.^10^ Barraza *et al* used a test-negative case-control approach to estimate vaccine effectiveness (VE) for a recent influenza season in Chile and found VE=49% for the reduction of hospitalizations based on 892 subjects (175 test-positive cases and 717 test-negative controls).^11^

In our analyses, female sex and subjects aged 65 years and older were more likely to be hospitalized for an influenza-related diagnosis in adults with or without diabetes. In the 2014 influenza season, we found a higher incidence of hospitalization events in women, potentially due to influenza A adversely affecting this population during this year.^23^ Both age and sex have been linked to altered outcomes with influenza infections, including higher levels of adverse events with older age and female sex, with age, sex, and influenza strain influencing these outcomes.^24 25^ Influenza vaccination earlier in the influenza season reduced the risk of these influenza events for subjects with diabetes regardless of age or sex category. In addition, though our study period covered the switch to high-dose vaccination in adults 65 years or older during the 2015–2016 influenza seasons,^26^ we did not observe noticeable changes in vaccine efficacy by our methodology. We observed that the risk reduction from vaccination or benefit provided by vaccination early in the influenza season was not significantly different for women than for men or subjects 65+ years.

Thus, this study provides clear evidence that all adults, but particularly patients with diabetes, should be encouraged to get the influenza vaccine at the start of the influenza season. Limitations of the study include the study population included in the provider systems may not be representative of other areas in the USA, our measure of influenza-related outcomes was estimated from specific ICD coding that may have over or underestimated these events, and the potential for confounding by factors such as functional status that was not fully addressed in our model approaches. In addition, our study time frame for the 2013–2016 influenza season, may not be representative of influenza infection and vaccine efficacy during individual years. Our analyses did not extend to the direct possible interactions of race and body mass index or other indices of adiposity regarding the reported effects on influenza infection-related comorbidities and the impact of influenza vaccination. We however controlled for this within comparisons made in our studies. Further analyses should be completed to directly examine the role of both diabetes (and measures of glycemic control) and obesity in regards to influenza-associated healthcare utilization in the future. Lastly, based on the data available in provider systems we were unable to directly compare vaccinated and unvaccinated subjects, and our analysis of relative risk of vaccination is focused on early versus late vaccination. In addition, using this data we have a risk of unmeasured confounding and selection bias. Regardless of these considerations, our findings provide quantitative data on the higher rates of healthcare utilization by subjects with diabetes with influenza infection. These studies support continued targeting of patients with diabetes for influenza vaccination campaigns, particularly early in the influenza season, in order to minimize severe disease outcomes and healthcare-associated costs.

## Supplementary material

10.1136/bmjdrc-2023-003841 online supplemental file 1

## Data Availability

Data may be obtained from a third party and are not publicly available.
