# Supplementary material for: Risk of healthcare visits from influenza in subjects with diabetes and impacts of early vaccination
Source: BMJ Open Diabetes Res Care. 2024 Aug 6;12(4):e003841. doi: 10.1136/bmjdrc-2023-003841 (PMC11308876; doi:10.1136/bmjdrc-2023-003841)
Supplement: online supplemental file 1 [file bmjdrc-12-4-s001.docx]

**Online only Supplemental Material For:**

**Risk of Healthcare Visits from Influenza in Subjects with Diabetes and Impacts of Early Vaccination.**

Ronald Horswell PhD^1^, San Chu MS^1^, Addison E. Stone BS^2^, Daniel Fort PhD^3^, Gabriel Uwaifo MD^4^, Vivian A. Fonseca MD^5^, Elizabeth B. Norton PhD^2*^

Affiliations:

^1^Pennington Biomedical Research Center, Louisiana State University, Baton Rouge, Louisiana

^2^Department of Microbiology and Immunology, Tulane University School of Medicine, New Orleans, Louisiana, USA

^3^Ochsner Center for Outcomes and Health Services Research, New Orleans, Louisiana, USA

^4^Ochsner Medical Center, Department of Endocrinology, Diabetes, and Metabolism, New Orleans, LA, USA

^5^Department of Medicine, Division of Endocrinology and Metabolism, Tulane University Health Sciences Center, School of Medicine, New Orleans, LA, United States, USA

**Online-Only Supplemental Contents:**

Methods

Table S1

Table S2

Table S3

Figure S1

Figure S2

Figure S3

Figure S4

Figure S5

**Supplemental Methods**

The primary analytical objective is to estimate the effect of influenza vaccination a defined target outcome for a particular population; for example, estimating the effect of vaccination on the level of all-cause inpatient admissions among diabetes patients. The analysis objectives also extend to comparing the magnitude of the vaccination effect between or among patient subgroups; for example, comparing the vaccination effect on all-cause admissions among diabetes patients to the effect among non-diabetes patients.

A “vaccination effect” might be defined in several alternative ways (i.e., by alternative effect metrics) depending on the intended subsequent uses of the analysis results and the nature of the outcome. Here we assume a “relative risk” metric is of interest, although much of the following would be relevant for other relative risk metrics as well.

Most importantly, we assume that the only data available to support the effect estimation are data derived from health care provider system’s electronic medical record (EMR) systems. Or, stated somewhat differently, we want a methodology appropriate for estimating influenza vaccination effects from EMR data.

Given the above objectives and context, however, several analysis issues arise, including:

1. Many cases of influenza resolve themselves without any contact with the health care system and, therefore, cannot be identified using EMR data or health care claims data.
2. Even for a particular target patient population whose clinical history is, in general, well-represented in the available analysis data set, many actual vaccination events may not be represented in the available EMR, a reality stemming from the diffuse vaccination locations now widely used for many types of vaccinations.
3. Within any flu season, there may be strong variability in the outcome level across calendar time; e.g., across months within a flu seasons. **Outcomes comparisons between vaccinated and non-vaccinated people must be made in some time-equivalent way, or the comparisons will be confounded with the across-time outcomes variation.** For example, outcomes during October for vaccinated individuals should not be compared with outcomes for January for unvaccinated individuals.
4. Within any flu season, there may be strong variability in the timing of vaccination events. This variability can lead to confounding of vaccination effects with outcomes variation across time.
5. Vaccination effectiveness may vary across time within a single flu season; e.g., across months within a single flu season.
6. An individual’s vaccination timing during flu season (e.g., month in which vaccination occurs) may be related to the individual’s probability of flu-related outcomes in ways not identifiable using the available data.
7. An individual’s vaccination may be motivated by immediately prior flu-related outcomes events, leading analytically to a potentially biasing “regression to the mean” phenomenon.
8. The immunity conferred by vaccination may be less for those vaccinated after having had the flu, as compared to those vaccinated prior to having had the flu.
9. The outcomes comparison of vaccinated to unvaccinated patients must be adjusted for demographics and for co-morbidities. But the number of co-morbidities is potentially quite large and may be context-specific.

We have developed two analytical approaches to estimating influenza vaccination effects using EMR data. The two approaches (which we call Methodology A and Methodology B) differ with regard to which of the above issues each methodology truly addresses.

**Overview of the Methodology A**. Methodology A functions by defining, for the flu season of interest, two groups, defined by differences in vaccination timing during the flu season, specifically, by whether vaccination occurred before versus after some “split date.” If, for example, December 31 is used as the split date, then for some particular flu season, the two groups would be defined as:

**Group 1:** those vaccinated at some point between September 1 and December 31 of the flu season.

**Group 2:** those vaccinated between January 1 and April 15 of the flu season.^[[1]](#footnote-1)^

Within each flu seasons, the methodology calls for forming matched pairs of subjects with one person from Group 1 matched to one person from Group 2 using as **matching criteria**:

1. Demographics (age, gender, race),
2. A comorbidity index based on data from before the flu season began (see Appendix A),
3. Number of outcomes events during the year immediately preceding the current flu season, and
4. Number of outcomes events during a particular (and subject-specific) early flu season time span called the “R” span (described below.)

Figure A depicts a timeline covering a hypothetical flu season. The timeline shows vaccination dates for two matched hypothetical individuals (a “Group 1 subject” and a “Group 2 subject”), both of whom were vaccinated during the flu season, but at different times. The “Group 1 subject” was vaccinated on or before the December 31 split date, while the “Group 2 subject” was vaccinated after December 31. The two subjects’ vaccination dates implicitly define three distinct time spans specific to the pair.

**Figure A**

Time span “R” is the time from September 1 to one week prior to the group 1 subject’s vaccination date.^[[2]](#footnote-2)^ **Span R is a time span during which neither of the two subjects has been vaccinated.**

Time span “S” is the time from two weeks after the group 1 subject’s vaccination date to one week before the group 2 subject’s vaccination date.^[[3]](#footnote-3)^ **Conceptually,** s**pan S is the time span within the flu season during which one of the two matched subjects was vaccinated, but the other was not.** Time span “T” is the time from two weeks after the group 2 subject’s vaccination to the end of flu season on April 15. **Conceptually, span T is a time when both of the matched subjects were vaccinated.**

Note that Span R not only is a characteristic of a matched pair, but also the number of outcome events during Span R is one of the “matching criteria” defined earlier. Operationally that implies that matched pairs are formed by iteratively matching two subjects based on matching criteria a, b, and c above and then accepting or rejecting each putative match depending on whether or not the pair also matches on number of outcome events in the implied Span R.

**Statistical Model for Methodology A.** Given the above Figure A design, estimation of the vaccination effect involves appropriately comparing the Group 1 and Group 2 matched subjects on outcomes over the aggregated “S” time spans. We also specify that only subjects with no outcomes events in their “R” spans are eligible for inclusion in the analysis. This last restriction helps reduce the potential confounding from several of the analysis issues listed earlier. An appropriate statistical model is then:

(Model A.1) E[Y_i,S_] = exp[β_0_ + β_1,S_V_i,S_ + log(person-years_i_) + φ_i_]

where

Y_i,S_ is the count of outcomes events for subject i over the subject i’s time span, S_i_,

β_0_ is the model intercept, in this context representing the aggregated outcomes rate of subjects who were non-vaccinated over their time spans, S_i,_

V_i,S_ is a binary indicator variable, with V_i,S_ = 0 for subjects non-vaccinated over their S_i_ time spans, and V_i,S_ = 1 for subjects vaccinated over their S_i_ time spans,

β_1,S_ is the relative outcomes risk for vaccination versus non-vaccination over the S_i_ time spans,

log(person-years_i,S_) is the model offset; i.e., in this context, the log of the person-years represented by each individual’s S_i_ time span, and

φ_i_ = an over-dispersion term, with exp(φ_i_) distributed as Γ(α,1/α).

Model A.1 might be estimated as a negative binomial model. However, in our actual estimation, we used Poisson regression with no over-dispersion term. Standard errors were derived by bootstrapping the entire estimation process, so as to account for various non-standard sources of variation, including any possible variance inflation due to over-dispersion.

The hypothesis test comparing the two groups is then:

H_0_: β_1,S_ = 0

versus H_1_: β_1,S_ ≠ 0

As specified above, Model A.1 estimates a vaccination effect within some particular group (e.g., among diabetes patients.) To compare the vaccination effects of two different groups (e.g., the vaccination effect among diabetes patient compared to the effect among non-diabetes patients), we expand Model A.1 to:

(Model A.2) E[Y_i,S_] = exp[β_0_ + β_1,SV_V_1i,S_ + β_2,SX_X_2i,SX_ + β_2,SV_V_2i,SV_ + log(person-years_i_) + φ_i_]

where now

Y_i,S_ is the count of outcomes events for subject i over the subject i’s time span, S_i_,

β_0_ is the model intercept, in this context representing the aggregated outcomes rate of group 1 subjects who were non-vaccinated over their time spans, S_i,_

V_1i,S_ is a binary indicator variable, with V_1i,S_ = 1 for group 1 subjects vaccinated over their S_i_ time spans, V_1i,S_ = 0 otherwise,

β_1,SV_ is a term capturing the effect of vaccination among group 1 subjects,

X_2i,SX_ is a binary indicator variable, with V_2i,SX_ = 1 for group 2 subjects not vaccinated over their S_i_ time spans, X_2i,SX_ = 0 otherwise,

β_2,SX_ is a term capturing the effect of non-vaccination among group 2 subjects,

V_2i,SV_ is a binary indicator variable, with V_2i,SV_ = 1 for group 2 subjects vaccinated over their S_i_ time spans, V_2i,SX_ = 0 otherwise,

β_2,SV_ is a term capturing the effect of vaccination among group 2 subjects,

log(person-years_i,S_) is the model offset; i.e., in this context, the log of the person-years represented by each individual’s S_i_ time span, and

φ_i_ = an over-dispersion term, with exp(φ_i_) distributed as Γ(α,1/α).

The hypothesis test comparing the vaccination effects of the two groups is then:

H_0_: β_2,SV_ – β_2,SX_ = β_1,SV_

versus H_1_: β_2,SV_ – β_2,SX_ = β_1,SV_

**Motivation for Methodology A and Key Assumptions.** The motivation for Methodology A stems largely from the first three analysis issues described in Box 1. Because outcomes rates vary considerably within any given flu season, Methodology A forms matched pairs in which, within each pair, outcomes are assessed over exactly the same calendar time span. In addition, the use of only subjects with known vaccination dates (i.e., vaccination dates identifiable in the available data) is intended to reduce bias that would be introduced if subjects without known vaccination dates were assumed to be non-vaccinated. The validity of Methodology A rests on three key underlying assumptions; specifically,

Assumption 1: **The timing of vaccinations for each member of a matched pair was unrelated to any actual occurrences of target outcomes events.** (Essentially, this is an assumption that the vaccination date for an included subject was not “triggered” by a recent actual occurrence of the target outcome.)

Assumption 2: **For the two subjects in each matched pair, the timing of vaccinations (e.g., which of the two was vaccinated first) was not related to any residual (i.e., after matching) difference between the two subjects in outcome risk.**

Assumption 3: **For the two subjects in each matched pair, relative risk reduction offered by vaccination does not vary over time during a flu season.**

Assumption 1 is essentially an assumption that Issue #7 (listed earlier) has no more than a negligible effect on the estimation of vaccination’s effect. Using only subjects with no “span R” outcomes events helps conform to this assumption.

Assumption 2 amounts to assuming Issue #6 is negligible, and Assumption 3 amounts to assuming Issue #5 is negligible. There is no *a priori* rationale implying Assumption 2 holds; actually, *a priori,* Assumption 2 seems unlikely to hold. Assumption 3 is perhaps *a priori* more reasonable, but still certainly not axiomatic. The most compelling reason for developing Methodology B (below) is to avoid the need for Assumptions 2 and 3; that is, to explicitly address Issues #5 and #6.

**2.5 Overview of the Methodology B**. The Methodology B data configuration differs considerably from that of Methodology A and is illustrated by Figure S3A which uses the 2016-17 flu season diabetes cohort as an example. The analysis includes only those diabetes patients with vaccination events represented in the available EMR data between October 1 of the flu season and the following March 31; in this example, between October 1, 2016 and March 31, 2017. The figure shows how the immunized fraction of that cohort changed from month to month during that flu season. [Note: The figure also includes September (with 100% non-vaccinated) and April (with 100% vaccinated) only to clarify that the cohort is explicitly defined to include only those who are known (from the available data) to have been vaccinated in the October 1 through March 31 time span.]^[[4]](#footnote-4)^

**Figure S3A**

Specifically, Figure S3A shows for each month the fraction of cohort’s person-years which were spent vaccinated and the fraction of person-years spent non-vaccinated. For example, in the data used to construct Figure S3A, the cohort for the 2016-17 flu season included 34,964 unique subjects; i.e., 34,964 people known to have been vaccinated sometime between October 1, 2016 and March 31, 2017.

Those 34,964 subjects spent (34,964/365)(31) = 2,970 person years in October 2016. As depicted in Figure S3A, during October, 89% of those 2,970 person-years was spent unvaccinated and 11% was spent vaccinated. However, in November, 48% of the cohort’s person-years was spent vaccinated, and 52% was spent non-unvaccinated, etc.

A key feature of Methodology B is that that each unique subject in a flu season cohort contributes person-years to all the months. Specifically, a subject contributes unvaccinated person-years to each month prior to the month of his vaccination and vaccinated person-years to each month after his month of vaccination. Typically, he contributes both unvaccinated and vaccinated person-years to the month during which his vaccination occurred.^[[5]](#footnote-5)^ The implication is that each subject acts as his/her own control, after adjusting for risk level, vaccination timing, and month.

**Model Specification for Methodology B.**  When implementing Methodology B, each subject becomes a “panel” with multiple observations stemming from the multiple time spans (e.g., multiple months) within a flu season. Specifically, when using “month” as the time span, each subject typically will have one observation for each month spent fully non-immunized, one observation for each month spent fully immunized, and two observations for the month during which the subject’s vaccination occurred. There are then several reasonable specifications for a Methodology B model, specifications which differ somewhat in how they address the various problematic analysis issues. Assuming that subjects have been stratified based on gender, race, and age group, the model specification is:

(Model B)

$E\left[ Y_{\mathrm{it}} \right]=exp{[\beta}_{0}+log(E_{it)}+\sum_{m} \varphi_{m}M_{m,it}+\sum_{m} \delta_{m}Q_{m,i}+\theta R_{i}+\lambda W_{i}+\beta_{T1}T_{1,it}+\phi P_{\mathrm{it}}+\beta_{T2}T_{2,it}+\beta_{T3}T_{3,it}]$

where

Y_m,it_ = the number of outcome events for subject i in time span t,

β_0_ is the model intercept capturing the effect for month 1 of flu season (October) when not vaccinated,

E_it_ = the exposure (in days) for subject i during time span t,

M_m,it_ =1 for an observation from month m, M_m,it_ = 0 otherwise,

ϕ_m_ captures the relative outcomes level in month m,

Q_m,it_ = 1 if subject i’s was vaccination occurred in month m, Q_m,it_ = 0 otherwise,

δ_m_ captures the relative outcomes risk attributable to those vaccinated in month m,

R_i_ is the comorbidity-related risk index value derived for subject i,

W_i_ = subject i’s number of outcomes events in the 12 months immediately prior to the flu season,

P_it_ = 1in the time period immediately prior to vaccination, P_it_ = 0 otherwise,

φ, λ, and φ capture the relative outcomes risk attributable to the R_i_ , W_i_, and P_it_ terms respectively.

T_1,it_ = 1 in any time period after subject i has been vaccinated if subject i had one or more outcomes events in previous time periods of the flu season, T_1,it_ = 0 otherwise,

T_2,it_ = 1 in any time period before subject i’s vaccination if subject i had no outcomes events in previous time periods of the flu season, T_2,it_ = 0 otherwise,

T_3,it_ = 1 in any time period after subject i’s vaccination if subject i had no outcomes events in previous time periods of the flu season, T_3,it_ = 0 otherwise,

β_T1_ captures the effect of vaccination on outcomes for subjects who had outcomes events in previous time periods of the flu season.

β_T2_ captures the effect of non-vaccination on outcomes for subjects who had no outcomes events in previous time periods of the flu season.

β_T3_ captures the effect of vaccination on outcomes for subjects who had no outcomes events in previous time periods of the flu season.

In this specification, the β_T1_T_1,it_, β_T2_T_1,it_, and β_T3_T_1,it_ terms provide for the possibility that the vaccination effect may differ depending on immunity acquired from prior outcomes events which may have involved influenza (see Issue # 8 in Table 1.) The relative benefit of vaccination among those who had flu season outcomes events prior to vaccination is exp(β_T1_), while the relative benefit of vaccination among those with no earlier outcomes events is modeled as exp(β_T2_)/ exp(β_T3_). That latter effect is of most interest, as it more closely represents the effect of vaccination apart from any immunity accrued from having the disease itself. Also, the large majority of subjects fall into the “no earlier outcomes events” category, so that exp(β_T2_)/ exp(β_T3_) is the effect of importance for the large majority of people. For that effect, the hypothesis test of interest is a test of the null hypothesis that exp(β_T2_)/ exp(β_T3_) = 1, or in the model’s original terms:

H_0_: β_T2_ - β_T3_ = 0

versus H_1_: β_T2_ - β_T3_ ≠ 0

The model can be expanded to support comparing two groups’ vaccination effects. Such an expansion simply involves a set of T terms for each of the two groups with the corresponding additional model parameters.

The Model B specification assumes that the data are stratified based on demographic variables and the observations are appropriately weighted. Specifically, data weights are created in a three-step process:

1. Define a target demographic stratum composition, such as the demographic composition of the overall sample.
2. Within each combination of month and immunization status, the data are weighted to reflect the relative strata proportions (in terms of person-years) of the overall sample.
3. The weights are then adjusted so that within each month, the sum of the weights across the vaccinated cases in the month equals the sum of the weights across the unvaccinated cases in the month.

**Motivation for Methodology B and Key Assumptions.** Methodology B is motivated by the desire to explicitly address certain of the analysis-related issues listed earlier. Specifically:

- The δ_m_Q_m,i_ terms are included to address for the possibility that month of vaccination may be related to risk in ways not discernable from known covariates; for example, those at relatively higher risk may be vaccinated earlier in the flu season (Issue #6.)
- The θR_i_ terms incorporate the comorbidity-related risk index level (Issue #9.)
- The λW_i_ portion of the model adjusts for actual outcomes event counts from the year just prior to the current flu season (also Issue #9.)
- The β_T1_T_1,i_ β_T2_T_1,i_ and β_T3_T_1,i_ terms provide for the possibility that the vaccination effect may differ depending on immunity acquired from prior outcomes events which may have involved influenza (Issue # 8.)

Whether or not the above specification $\mathrm{provides}$unbiased estimation of the vaccination versus non-vaccination risk ratio depends on the how well the above included terms truly adjust for their target analysis issues. Further, this particular specification does not explicitly address Issue #5 in Box 1. As a result, the Model B specification assumes that (a) the vaccination effect is uniform over the months within a flu season, or (b) only the average vaccination effect across a particular flu season’s months is of interest.

The question arises, could the need for the assumption of effect uniformity across months be avoided by replacing using individual terms for vaccination status for each month? That can be done, but other aspects of the model also would have to be re-parameterized to avoid confounding. However, the assumption of uniformity of the vaccination effect across months can be tested.

**Adjustment for Comorbidities and Outcomes History:** As described above, Models A.1 and A.2 involve created matched pairs of subjects. A comorbidity index is among the variables used in that pairwise matching. In Model B, the comorbidity index is incorporated as an explicit covariate in the statistical model. The practical difficulty of matching subjects on numerous comorbidities has led to creation of various comorbidity indexes. However, the need here (and in most contexts) is for a comorbidity index constructed to be directly relevant to the target population and the target outcomes of interest. The large number of potential comorbidities complicates creation of such an index. However, the fundamental need is not that the vaccinated and non-vaccinated groups be equated on all comorbidities. Rather **the need is to equate the two groups on comorbidity-related probabilities of the target outcome events**. To enable this, we use the following approach:

- We define comorbidities based on three-digit ICD-9 and ICD-10 codes; i.e., the first three digits of an ICD-9 or ICD-10 diagnosis code. Here we refer to those as “three-digit comorbidities.”
- For any particular flu season’s analysis, we consider a subject to have a particular pre-existing comorbidity, if the subject’s diagnosis codes over the year prior to the flu season include the three-digit comorbidity.
- For each three-digit comorbidity, we estimate, in a separate exercise, the relative probability of the target outcomes events associated with that three-digit comorbidity.
- For each subject, we summed those probabilities over the subject’s three-digit comorbidities, and that sum was used as a measure of relevant comorbidity burden.

The above is not intended to achieve matching on all comorbidities or even on specific comorbidities. Rather the purpose of the created index is to enable matching on the aggregate comorbidity-related probability of the target outcomes

**Table S1**.  **ICD-9 and ICD-10 Diagnostic Codes Used for Influenza Categories.** Iped_Infpneu codes were used to quantify influenza events and Iped_allresp used for respiratory events.

| **system** | **dx** | **iped_infpneu** | **influenza viral or non-specific pneumonia** | **influenza only** | **iped_resp** | **concept_name** |
| --- | --- | --- | --- | --- | --- | --- |
| ICD-9-CM | 487 | infpneu | influenza_readj | influenza | allresp | Influenza |
| ICD-9-CM | 488 | infpneu | influenza_readj | influenza | allresp | Influenza due to certain identified influenza viruses |
| ICD-10-CM | J09 | infpneu | influenza_readj | influenza | allresp | Influenza due to certain identified influenza viruses |
| ICD-10-CM | J10 | infpneu | influenza_readj | influenza | allresp | Influenza due to other identified influenza virus |
| ICD-10-CM | J11 | infpneu | influenza_readj | influenza | allresp | Influenza due to unidentified influenza virus |
| ICD-9-CM | 480 | infpneu | influenza_readj |  | allresp | Viral pneumonia |
| ICD-9-CM | 483 | infpneu | influenza_readj |  | allresp | Pneumonia due to other specified organism |
| ICD-9-CM | 484 | infpneu | influenza_readj |  | allresp | Pneumonia in infectious diseases classified elsewhere |
| ICD-9-CM | 485 | infpneu | influenza_readj |  | allresp | Bronchopneumonia, organism unspecified |
| ICD-9-CM | 486 | infpneu | influenza_readj |  | allresp | Pneumonia, organism unspecified |
| ICD-10-CM | J12 | infpneu | influenza_readj |  | allresp | Viral pneumonia, not elsewhere classified |
| ICD-10-CM | J16 | infpneu | influenza_readj |  | allresp | Pneumonia due to other infectious organisms, not elsewhere classified |
| ICD-10-CM | J17 | infpneu | influenza_readj |  | allresp | Pneumonia in diseases classified elsewhere |
| ICD-10-CM | J18 | infpneu | influenza_readj |  | allresp | Pneumonia, unspecified organism |
| ICD-9-CM | 481 | infpneu |  |  | allresp | Pneumococcal pneumonia [Streptococcus pneumoniae pneumonia] |
| ICD-9-CM | 482 | infpneu |  |  | allresp | Other bacterial pneumonia |
| ICD-10-CM | J13 | infpneu |  |  | allresp | Pneumonia due to Streptococcus pneumoniae |
| ICD-10-CM | J14 | infpneu |  |  | allresp | Pneumonia due to Hemophilus influenzae |
| ICD-10-CM | J15 | infpneu |  |  | allresp | Bacterial pneumonia, not elsewhere classified |
| ICD-9-CM | 460 |  |  |  | allresp | Acute nasopharyngitis [common cold] |
| ICD-9-CM | 461 |  |  |  | allresp | Acute sinusitis |
| ICD-9-CM | 462 |  |  |  | allresp | Acute pharyngitis |
| ICD-9-CM | 463 |  |  |  | allresp | Acute tonsillitis |
| ICD-9-CM | 464 |  |  |  | allresp | Acute laryngitis and tracheitis |
| ICD-9-CM | 465 |  |  |  | allresp | Acute upper respiratory infections of multiple or unspecified sites |
| ICD-9-CM | 466 |  |  |  | allresp | Acute bronchitis and bronchiolitis |
| ICD-9-CM | 470 |  |  |  | allresp | Deviated nasal septum |
| ICD-9-CM | 471 |  |  |  | allresp | Nasal polyps |
| ICD-9-CM | 472 |  |  |  | allresp | Chronic pharyngitis and nasopharyngitis |
| ICD-9-CM | 473 |  |  |  | allresp | Chronic sinusitis |
| ICD-9-CM | 474 |  |  |  | allresp | Chronic disease of tonsils and adenoids |
| ICD-9-CM | 475 |  |  |  | allresp | Peritonsillar abscess |
| ICD-9-CM | 476 |  |  |  | allresp | Chronic laryngitis and laryngotracheitis |
| ICD-9-CM | 477 |  |  |  | allresp | Allergic rhinitis |
| ICD-9-CM | 478 |  |  |  | allresp | Other diseases of upper respiratory tract |
| ICD-9-CM | 490 |  |  |  | allresp | Bronchitis, not specified as acute or chronic |
| ICD-9-CM | 491 |  |  |  | allresp | Chronic bronchitis |
| ICD-9-CM | 492 |  |  |  | allresp | Emphysema |
| ICD-9-CM | 493 |  |  |  | allresp | Asthma |
| ICD-9-CM | 494 |  |  |  | allresp | Bronchiectasis |
| ICD-9-CM | 495 |  |  |  | allresp | Extrinsic allergic alveolitis |
| ICD-9-CM | 496 |  |  |  | allresp | Chronic airway obstruction, not elsewhere classified |
| ICD-9-CM | 500 |  |  |  | allresp | Coal workers' pneumoconiosis |
| ICD-9-CM | 501 |  |  |  | allresp | Asbestosis |
| ICD-9-CM | 502 |  |  |  | allresp | Pneumoconiosis due to other silica or silicates |
| ICD-9-CM | 503 |  |  |  | allresp | Pneumoconiosis due to other inorganic dust |
| ICD-9-CM | 504 |  |  |  | allresp | Pneumonopathy due to inhalation of other dust |
| ICD-9-CM | 505 |  |  |  | allresp | Pneumoconiosis, unspecified |
| ICD-9-CM | 506 |  |  |  | allresp | Respiratory conditions due to chemical fumes and vapors |
| ICD-9-CM | 507 |  |  |  | allresp | Pneumonitis due to solids and liquids |
| ICD-9-CM | 508 |  |  |  | allresp | Respiratory conditions due to other and unspecified external agents |
| ICD-9-CM | 510 |  |  |  | allresp | Empyema |
| ICD-9-CM | 511 |  |  |  | allresp | Pleurisy |
| ICD-9-CM | 512 |  |  |  | allresp | Pneumothorax and air leak |
| ICD-9-CM | 513 |  |  |  | allresp | Abscess of lung and mediastinum |
| ICD-9-CM | 514 |  |  |  | allresp | Pulmonary congestion and hypostasis |
| ICD-9-CM | 515 |  |  |  | allresp | Postinflammatory pulmonary fibrosis |
| ICD-9-CM | 516 |  |  |  | allresp | Other alveolar and parietoalveolar pneumonopathy |
| ICD-9-CM | 517 |  |  |  | allresp | Lung involvement in conditions classified elsewhere |
| ICD-9-CM | 518 |  |  |  | allresp | Other diseases of lung |
| ICD-9-CM | 519 |  |  |  | allresp | Other diseases of respiratory system |
| ICD-10-CM | J00 |  |  |  | allresp | Acute nasopharyngitis [common cold] |
| ICD-10-CM | J01 |  |  |  | allresp | Acute sinusitis |
| ICD-10-CM | J02 |  |  |  | allresp | Acute pharyngitis |
| ICD-10-CM | J03 |  |  |  | allresp | Acute tonsillitis |
| ICD-10-CM | J04 |  |  |  | allresp | Acute laryngitis and tracheitis |
| ICD-10-CM | J05 |  |  |  | allresp | Acute obstructive laryngitis [croup] and epiglottitis |
| ICD-10-CM | J06 |  |  |  | allresp | Acute upper respiratory infections of multiple and unspecified sites |
| ICD-10-CM | J20 |  |  |  | allresp | Acute bronchitis |
| ICD-10-CM | J21 |  |  |  | allresp | Acute bronchiolitis |
| ICD-10-CM | J22 |  |  |  | allresp | Unspecified acute lower respiratory infection |
| ICD-10-CM | J30 |  |  |  | allresp | Vasomotor and allergic rhinitis |
| ICD-10-CM | J31 |  |  |  | allresp | Chronic rhinitis, nasopharyngitis and pharyngitis |
| ICD-10-CM | J32 |  |  |  | allresp | Chronic sinusitis |
| ICD-10-CM | J33 |  |  |  | allresp | Nasal polyp |
| ICD-10-CM | J34 |  |  |  | allresp | Other and unspecified disorders of nose and nasal sinuses |
| ICD-10-CM | J35 |  |  |  | allresp | Chronic diseases of tonsils and adenoids |
| ICD-10-CM | J36 |  |  |  | allresp | Peritonsillar abscess |
| ICD-10-CM | J37 |  |  |  | allresp | Chronic laryngitis and laryngotracheitis |
| ICD-10-CM | J38 |  |  |  | allresp | Diseases of vocal cords and larynx, not elsewhere classified |
| ICD-10-CM | J39 |  |  |  | allresp | Other diseases of upper respiratory tract |
| ICD-10-CM | J40 |  |  |  | allresp | Bronchitis, not specified as acute or chronic |
| ICD-10-CM | J41 |  |  |  | allresp | Simple and mucopurulent chronic bronchitis |
| ICD-10-CM | J42 |  |  |  | allresp | Unspecified chronic bronchitis |
| ICD-10-CM | J43 |  |  |  | allresp | Emphysema |
| ICD-10-CM | J44 |  |  |  | allresp | Other chronic obstructive pulmonary disease |
| ICD-10-CM | J45 |  |  |  | allresp | Asthma |
| ICD-10-CM | J47 |  |  |  | allresp | Bronchiectasis |
| ICD-10-CM | J60 |  |  |  | allresp | Coalworker's pneumoconiosis |
| ICD-10-CM | J61 |  |  |  | allresp | Pneumoconiosis due to asbestos and other mineral fibers |
| ICD-10-CM | J62 |  |  |  | allresp | Pneumoconiosis due to dust containing silica |
| ICD-10-CM | J63 |  |  |  | allresp | Pneumoconiosis due to other inorganic dusts |
| ICD-10-CM | J64 |  |  |  | allresp | Unspecified pneumoconiosis |
| ICD-10-CM | J65 |  |  |  | allresp | Pneumoconiosis associated with tuberculosis |
| ICD-10-CM | J66 |  |  |  | allresp | Airway disease due to specific organic dust |
| ICD-10-CM | J67 |  |  |  | allresp | Hypersensitivity pneumonitis due to organic dust |
| ICD-10-CM | J68 |  |  |  | allresp | Respiratory conditions due to inhalation of chemicals, gases, fumes and vapors |
| ICD-10-CM | J69 |  |  |  | allresp | Pneumonitis due to solids and liquids |
| ICD-10-CM | J70 |  |  |  | allresp | Respiratory conditions due to other external agents |
| ICD-10-CM | J80 |  |  |  | allresp | Acute respiratory distress syndrome |
| ICD-10-CM | J81 |  |  |  | allresp | Pulmonary edema |
| ICD-10-CM | J82 |  |  |  | allresp | Pulmonary eosinophilia, not elsewhere classified |
| ICD-10-CM | J84 |  |  |  | allresp | Other interstitial pulmonary diseases |
| ICD-10-CM | J85 |  |  |  | allresp | Abscess of lung and mediastinum |
| ICD-10-CM | J86 |  |  |  | allresp | Pyothorax |
| ICD-10-CM | J90 |  |  |  | allresp | Pleural effusion, not elsewhere classified |
| ICD-10-CM | J91 |  |  |  | allresp | Pleural effusion in conditions classified elsewhere |
| ICD-10-CM | J92 |  |  |  | allresp | Pleural plaque |
| ICD-10-CM | J93 |  |  |  | allresp | Pneumothorax and air leak |
| ICD-10-CM | J94 |  |  |  | allresp | Other pleural conditions |
| ICD-10-CM | J95 |  |  |  | allresp | Intraoperative and postprocedural complications and disorders of respiratory system,  not elsewhere classified |
| ICD-10-CM | J96 |  |  |  | allresp | Respiratory failure, not elsewhere classified |
| ICD-10-CM | J98 |  |  |  | allresp | Other respiratory disorders |
| ICD-10-CM | J99 |  |  |  | allresp | Respiratory disorders in diseases classified elsewhere |

**Table S2. Subject demographics by flu season and diabetes history**

| Flu Season Subjects from September - April (starting year) | | | | | | | | | | | |
| --- | --- | --- | --- | --- | --- | --- | --- | --- | --- | --- | --- |
|  |  | **2012** | | **2013** | | **2014** | | **2015** | | **2016** | |
| **Diabetes history** | | **no** | **yes** | **no** | **yes** | **no** | **yes** | **no** | **yes** | **no** | **yes** |
| **Subjects** | **no. (%)** | 89,139 (65%) | 48,308 (35%) | 219,662 (70%) | 94,953 (30%) | 254,728 (70%) | 109,297 (30%) | 267,272 (70%) | 117,252 (30%) | 285,916 (70%) | 126,427 (30%) |
|  |  |  |  |  |  |  |  |  |  |  |  |
| **Female** | **no. (%)** | 59,745 (67%) | 27,027 (56%) | 143,088 (65%) | 53,513 (56%) | 164,860 (65%) | 61,597 (56%) | 171,916 (64%) | 65,569 (56%) | 183,851 (64%) | 68,876 (56%) |
|  |  |  |  |  |  |  |  |  |  |  |  |
| **Asian** | **no. (%)** | 835 (1%) | 365 (1%) | 2,638 (1%) | 724 (1%) | 3 (1%) | 868 (1%) | 3,858 (1%) | 1,005 (1%) | 4,361 (2%) | 1,143 (1%) |
| **Black** | **no. (%)** | 25,184 (28%) | 16,524 (34%) | 62,515 (28%) | 33,293 (35%) | 73,914 (29%) | 39,285 (36%) | 77,153 (29%) | 42,383 (36%) | 82,448 (29%) | 44,927 (36%) |
| **White** | **no. (%)** | 62,098 (70%) | 30,989 (64%) | 151,568 (69%) | 60,049 (63%) | 173,720 (68%) | 68,121 (62%) | 181,836 (68%) | 72,692 (62%) | 193,845 (68%) | 76,047 (62%) |
| **Other race** | **no. (%)** | 1,022 (1%) | 430 (1%) | 2,941 (1%) | 884 (1%) | 3,733 (1%) | 1,029 (1%) | 4,425 (2%) | 1,172 (1%) | 5,262 (2%) | 1,310 (1%) |
|  |  |  |  |  |  |  |  |  |  |  |  |
| **Hispanic** | **no. (%)** | 1,689 (2%) | 909 (2%) | 5,165 (2%) | 1,950 (2%) | 6,373 (3%) | 2,253 (2%) | 6,760 (3%) | 2,459 (2%) | 7,585 (3%) | 2,592 (2%) |
|  |  |  |  |  |  |  |  |  |  |  |  |
| **Age** | **min** | 18 | 18 | 18 | 18 | 18 | 18 | 18 | 18 | 18 | 18 |
|  | **max** | 90 | 90 | 90 | 90 | 90 | 90 | 90 | 90 | 90 | 90 |
|  | **median** | 52 | 64 | 48 | 63 | 48 | 63 | 49 | 63 | 49 | 63 |
|  | **>65 no. (%)** | 21,772 (24%) | 23,690 (49%) | 44,170 (20%) | 43,598 (46%) | 51,398 (20%) | 50,245 (46%) | 55,696 (21%) | 54,468 (46%) | 61,026 (21%) | 58,185 (47%) |

**Table S2. Influenza event rate (iped_infpneu) per person by diabetic status with 95% confidence intervals and unadjusted/adjusted comparisons by flu year and averages.**

|  |  |  |  |  |  |  |  | ***Unadjusted*** |  |  |  | ***Adjusted*** |  |  |  |  |  |  |
| --- | --- | --- | --- | --- | --- | --- | --- | --- | --- | --- | --- | --- | --- | --- | --- | --- | --- | --- |
|  |  |  | ***Diabetes Patients*** | | |  | ***non-Diabetes Patients*** | | |  | ***non-Diabetes Patients*** | | |  |  |  | event rate comparisons | |
| **outcome** | **fluyear** |  | **event rate** | **lower** | **upper** |  | **event rate** | **lower** | **upper** |  | **event rate** | **lower** | **upper** |  | all events | | **non-adj** | **adj** |
| iped_infpneu | 2013 |  | 0.0143 | 0.0134 | 0.0152 |  | 0.0026 | 0.0023 | 0.0029 |  | 0.0033 | 0.0029 | 0.0036 |  | 0.0169 |  | 5.5 | 4.4 |
| iped_infpneu | 2014 |  | 0.0141 | 0.0133 | 0.0150 |  | 0.0024 | 0.0021 | 0.0027 |  | 0.0040 | 0.0036 | 0.0044 |  | 0.0165 |  | 5.9 | 3.5 |
| iped_infpneu | 2015 |  | 0.0106 | 0.0099 | 0.0113 |  | 0.0016 | 0.0014 | 0.0018 |  | 0.0022 | 0.0019 | 0.0024 |  | 0.0122 |  | 6.5 | 4.9 |
| iped_infpneu | 2016 |  | 0.0137 | 0.0130 | 0.0146 |  | 0.0028 | 0.0025 | 0.0031 |  | 0.0049 | 0.0045 | 0.0053 |  | 0.0166 |  | 4.9 | 2.8 |
|  | average |  | 0.0132 |  |  |  | 0.0024 |  |  |  | 0.0036 |  |  |  | 0.0155 |  | 5.6 | 3.7 |

**Table S3. Relative Risk values for each model (all years) for immunized vs. non-immunized by diabetes status with with 95% confidence intervals and associated P-values.**

**Supplemental Figures**

**
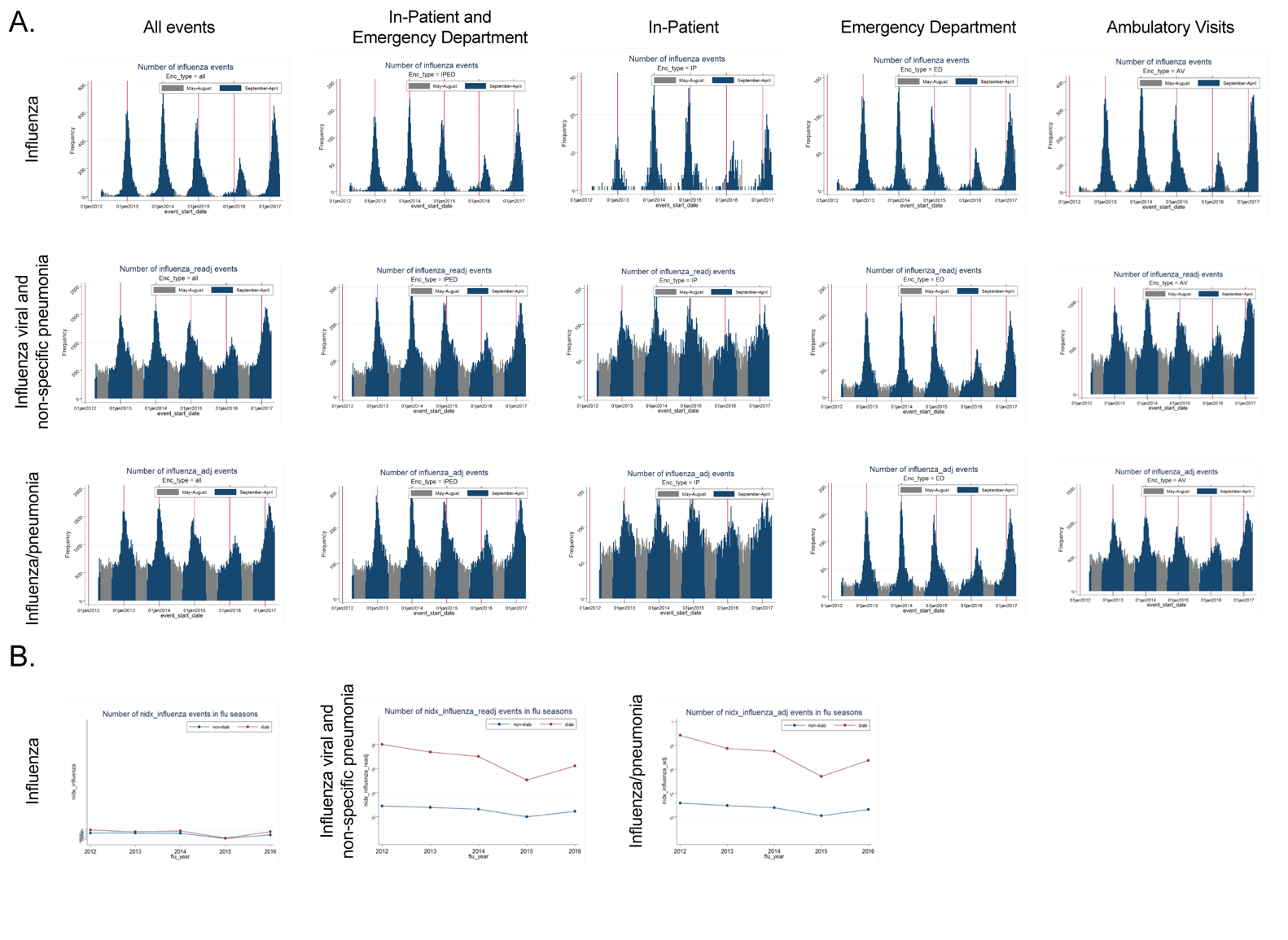
**

**Figure S1. Seasonality in influenza events regardless of disease classification codes.** (A) Number of all events, in-patient and emergency department, in-patient, emergency department, and ambulatory visits using general influenza definition, influenza viral and non-specific pneumonia, and influenza-specific pneumonia parameters across flu seasons. (B) Number of emergency department and in-patient events for general influenza definition, influenza viral and non-specific pneumonia, and influenza-specific pneumonia parameters across flu seasons in patients with or without diabetes.

**Figure S2. Strategy for Identifying Records used for immunization analyses.**

(The numbers in blue are unique subjects within a flu season.)


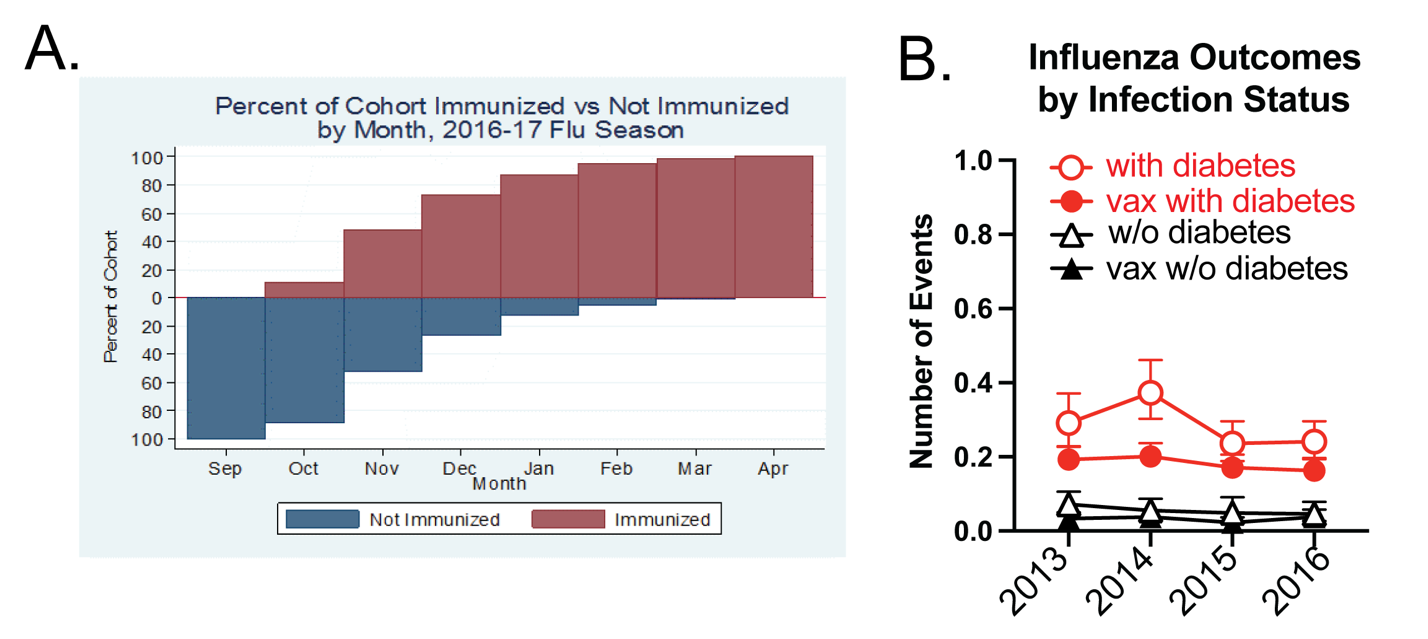


**Figure S3. Immunization History and Influenza Events from Model 2 Analyses.** (A) Proportion of study cohort immunization across 2016-2017 influenza season. (B) Influenza related hospitalization in subjects with or without diabetes by vaccination history using ‘matched strata’ model 2 analysis.

**
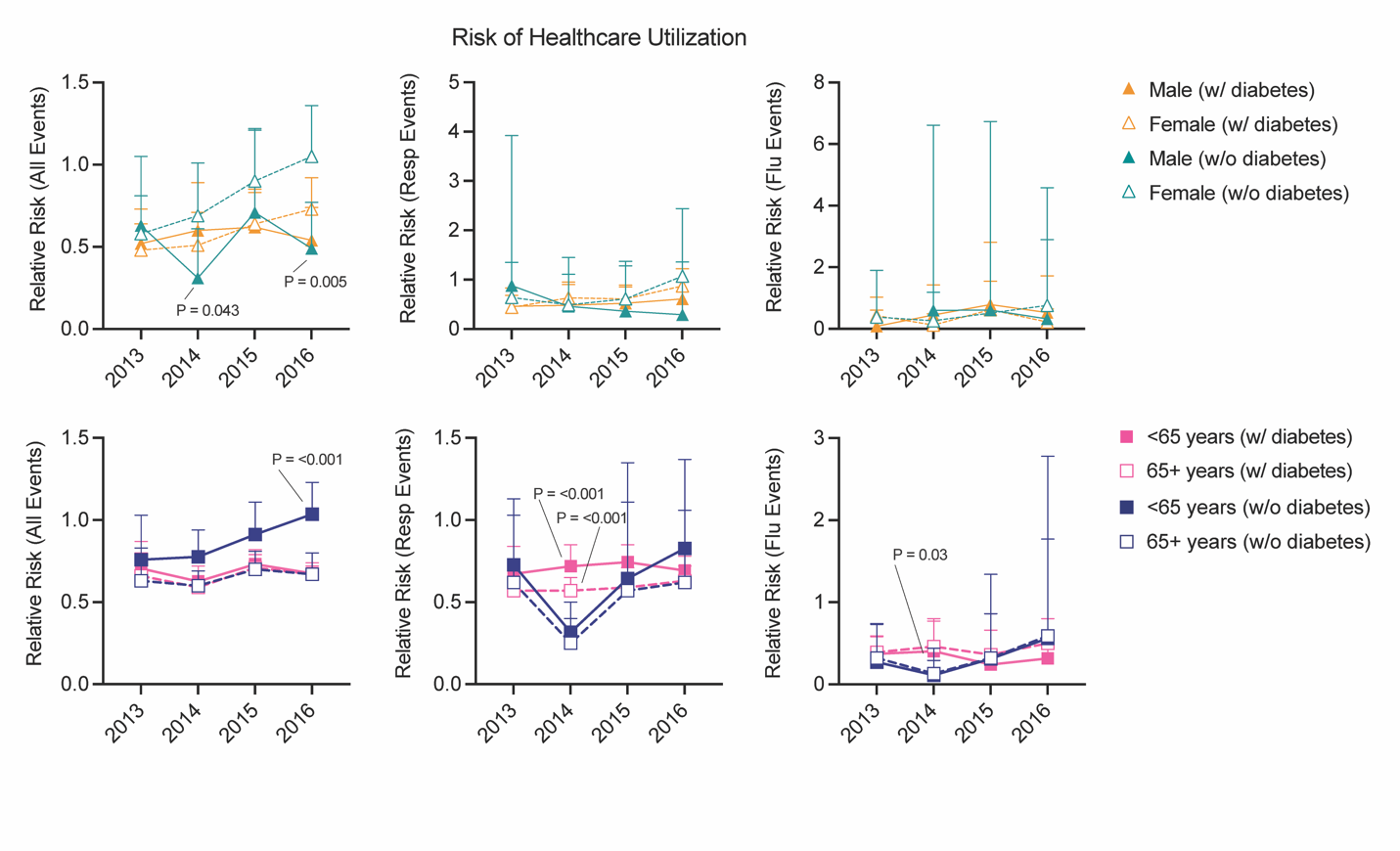
**

**Figure S4. Age and Gender Differences in Relative Risk of Healthcare Utilization.** Relative risk of all, respiratory, and influenza related visits in immunized vs non-immunized subjects. Top row: Relative risk by sex and diabetes diagnosis by flu season. Statistical comparisons for male and female comparisons within and between patient groups (e.g. subjects with or without diabetes) were not significant except for all events observed in the 2014 and 2016 flu seasons for the male and female comparison in subjects without diabetes as indicated (this was not significant for subjects with diabetes). No significance was found in any comparisons made for respiratory or flu events or for all flu seasons. Bottom row: Relative risk by age category and diabetes diagnosis by flu season. Statistical comparisons for age group within and between patient groups for all years were not significant, but for individual years are indicated, including 2016 flu season risk of all cause visits between subjects with and without diabetes P<0.001 for <65 years age group; 2014 flu season risk of respiratory related visit between subjects with and without diabetes P<0.001 in both 65+ years and <65 years age group; and lastly 2014 flu season risk of influenza related visit between subjects with and without diabetes P<0.03 for <65 years age group. Error bars indicate 95% confidence intervals.


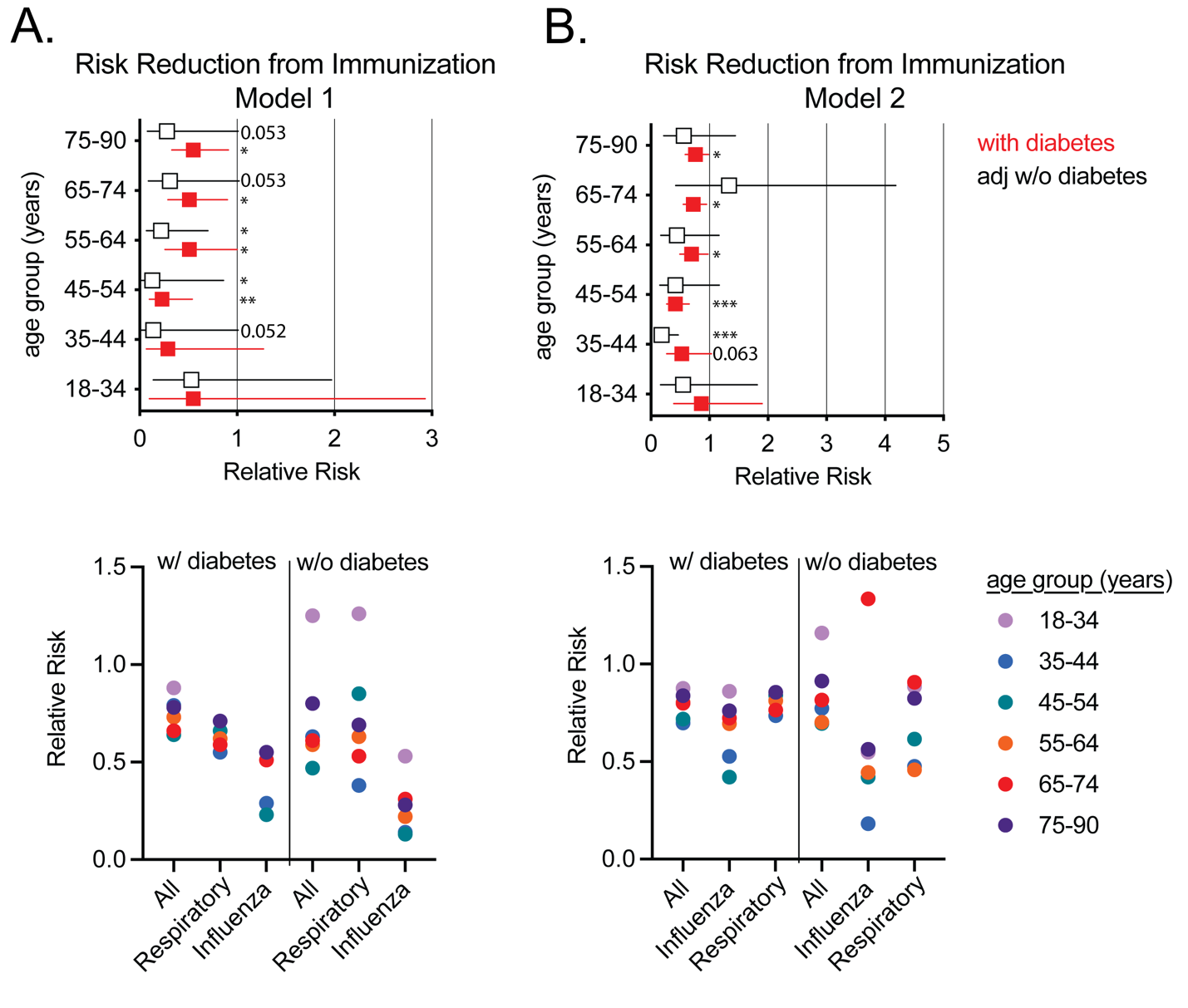


**Figure S5. Relative risk of healthcare utilization by age group.** (A) Risk reduction from immunization by age group using Model 1. (B) Risk reduction from immunization by age group using Model 2. P-values indicated as written of coded as * P<0.05, ** P< 0.01, *** P<0.001. Error bars indicate 95% confidence intervals.

1. The December 31 “split date” defines membership in Groups 1 and 2. However, other “split dates” during a flu season could be used instead. December 31 is simply a convenient date that splits the flu season roughly in half. [↑](#footnote-ref-1)
2. Here we depict span R as beginning on September 1, but that beginning date could be extended back somewhat further, such as to August 1. [↑](#footnote-ref-2)
3. The reasons for the one-week window before vaccination and the two-week window after vaccination are discussed in a later section. [↑](#footnote-ref-3)
4. Methodology B requires dividing the span between October 1 and March 31 into time intervals, but the intervals do not have to be months or even of roughly equal length. It is important, however, that the time span covered by each interval not be too long. [↑](#footnote-ref-4)
5. Some individuals contribute only unvaccinated or vaccinated person-years to the month of vaccination, because for bias avoidance reasons, the week before and the two weeks after vaccination are dropped for each subject. Those weeks also are dropped when counting outcomes. [↑](#footnote-ref-5)
